# Variation in DNA Methylation in Avian Nestlings Is Largely Determined by Genetic Effects

**DOI:** 10.1093/molbev/msad086

**Published:** 2023-04-11

**Authors:** Bernice Sepers, Rebecca Shuhua Chen, Michelle Memelink, Koen J F Verhoeven, Kees van Oers

**Affiliations:** Department of Animal Ecology, Netherlands Institute of Ecology (NIOO-KNAW), Wageningen, The Netherlands; Behavioural Ecology Group, Wageningen University & Research (WUR), Wageningen, The Netherlands; Department of Animal Behaviour, University of Bielefeld, Bielefeld, Germany; Department of Animal Ecology, Netherlands Institute of Ecology (NIOO-KNAW), Wageningen, The Netherlands; Department of Animal Behaviour, University of Bielefeld, Bielefeld, Germany; Department of Animal Ecology, Netherlands Institute of Ecology (NIOO-KNAW), Wageningen, The Netherlands; Department of Terrestrial Ecology, Netherlands Institute of Ecology (NIOO-KNAW), Wageningen, The Netherlands; Department of Animal Ecology, Netherlands Institute of Ecology (NIOO-KNAW), Wageningen, The Netherlands; Behavioural Ecology Group, Wageningen University & Research (WUR), Wageningen, The Netherlands

**Keywords:** epigenetics, DNA methylation, vertebrate, mQTL, cross-fostering

## Abstract

As environmental fluctuations are becoming more common, organisms need to rapidly adapt to anthropogenic, climatic, and ecological changes. Epigenetic modifications and DNA methylation in particular provide organisms with a mechanism to shape their phenotypic responses during development. Studies suggest that environmentally induced DNA methylation might allow for adaptive phenotypic plasticity that could last throughout an organism's lifetime. Despite a number of studies demonstrating environmentally induced DNA methylation changes, we know relatively little about what proportion of the epigenome is affected by environmental factors, rather than being a consequence of genetic variation. In the current study, we use a partial cross-foster design in a natural great tit (*Parus major*) population to disentangle the effects of common origin from common rearing environment on DNA methylation. We found that variance in DNA methylation in 8,315 CpG sites was explained by a common origin and only in 101 by a common rearing environment. Subsequently, we mapped quantitative trait loci for the brood of origin CpG sites and detected 754 *cis* and 4,202 *trans* methylation quantitative trait loci, involving 24% of the CpG sites. Our results indicate that the scope for environmentally induced methylation marks independent of the genotype is limited and that the majority of variation in DNA methylation early in life is determined by genetic factors instead. These findings suggest that there may be little opportunity for selection to act on variation in DNA methylation. This implies that most DNA methylation variation likely does not evolve independently of genomic changes.

## Introduction

Due to climate change and urbanization, the natural environment is changing rapidly. Phenotypic plasticity, the process in which a genotype produces a variety of phenotypes under varying environmental conditions (Pigliucci 2001), is expected to play a crucial role when organisms attempt to adapt to those changes. Epigenetic modifications are mechanisms that regulate gene expression without changing the primary nucleotide sequence (Richards 2006), and they are expected to underlie phenotypic plasticity shaped by developmental processes (Pigliucci 2001). However, we lack information on the origin of these epigenetic modifications and how they may play a role in adaptive changes to rapid environmental changes (Yona et al. 2015; Liu et al. 2020).

One of the best-studied epigenetic mechanisms is DNA methylation, which involves the attachment of a methyl group to, most commonly in vertebrates and insects, a cytosine base pair (Auclair and Weber 2012). In vertebrates, especially for DNA methylation in a CpG context, a cytosine followed by a guanine nucleotide in the 5′–3′ direction is known to regulate gene expression (Feng et al. 2010). These CpG sites (CpGs) tend to cluster in CpG islands around promoter regions and transcription start sites (TSS) (Usui et al. 2009; Li 2011; Jones 2012) and are generally found to be hypomethylated at promoters or in intergenic regions but hypermethylated in gene bodies (Feng et al. 2010; Deaton and Bird 2011; Laine et al. 2016). CpG island methylation regulates gene expression during development (Derks et al. 2016), while it also has the ability to respond to environmental cues across a variety of organisms (Gugger et al. 2016; Lea et al. 2016; Sheldon et al. 2018; Venkataraman et al. 2020; Sepers et al. 2021; Venney et al. 2021). Moreover, DNA methylation marks are mitotically heritable, meaning that alterations can persist throughout an individual's lifetime through cell division, and to some extent, they are hypothesized to span multiple generations (Heard and Martienssen 2014; Nagy and Turecki 2015; Otterdijk and Michels 2016; Guerrero-Bosagna et al. 2018; Perez and Lehner 2019). Therefore, DNA methylation has a great potential to explain phenotypic plasticity from a proximate perspective.

Various factors can impact the methylation state of a CpG site. First of all, a proportion of methylation marks is highly genetically programmed, and little to no variation is observed between individuals (Razin and Szyf 1984). Such marks are important for regulating cellular differentiation and organogenesis: processes that bring rise to cell identity (Richards 2006). Interindividual variation in DNA methylation can be dependent on genetic variation and spontaneous mutations and/or can be environmentally induced (Sepers et al. 2019). Genetic variation can lead to certain epigenetic states, for example, when a particular sequence variation generates a specific epigenetic mark. There can be marks that are solely dependent on the genotype, regardless of environmental cues, called obligatory marks (Richards 2006). Facilitated marks are to some extent limited to the genotype, such as when a certain mutation or allele leads to an altered or loss of protein function (Richards 2006). In animals, DNA methylation is catalyzed by DNA methyltransferases (*DNMTs*) and methyltransferase-like proteins (*METTLs*) (Cheng and Blumenthal 1999; Robertson and Wolffe 2000). Although the diversification of these enzymes in terms of structure and function across taxa is not fully understood, *DNMTs* are known to be important for both de novo establishment and maintenance of DNA methylation (Okano et al. 1999; Goll and Bestor 2005; Cheng and Blumenthal 2008; Kim et al. 2009). *METLLs* have not received as much attention as *DNMTs*, but their function seems to strongly depend on the *METTL* type. Some types modify RNA and proteins, while others modify DNA (for an overview, see Wong and Eirin-Lopez 2021). Therefore, genetic variation in at least some of the genes encoding those enzymes, especially *DNMTs*, is expected to have genome-wide effects on DNA methylation.

In addition to a genetic contribution, interindividual variation in DNA methylation can also be environmentally induced. Environmental cues may give rise to changes in epigenetic states, leading to phenotypic differences within the lifetime of an individual. In an absence of genetic factors on methylation states at a particular mark, this is referred to as a pure epigenetic mark (Richards 2006). DNA methylation is especially prone to changes during early life. In vertebrates, DNA methylation is reported to be affected during early life by temperature (Sheldon et al. 2020), the type of rearing environment (Morán et al. 2013; Venney et al. 2021), brood size (Sheldon et al. 2018; Sepers et al. 2021), parental care (Weaver et al. 2004), nutrition (Waterland and Jirtle 2003; Sandovici et al. 2011), and pollutant exposure (Laine et al. 2021; Mäkinen et al. 2022). Thus, methylation marks can either be highly programmed and differ little in state between individuals or be influenced by a combination of genetic and environmental factors that lead to inter- and intraindividual variation in DNA methylation states.

However, despite evidence showing phenotypic changes in response to environmental cues, knowledge is limited on the extent of the plasticity of the epigenome, including the proportion of the epigenome that is determined by genetic influences as opposed to environmental cues. Moreover, epigenetic marks that are environmentally induced are hypothesized to facilitate genetic change when inherited transgenerationally independent of the genotype (Jablonka and Raz 2009; Guerrero-Bosagna et al. 2018). One method to assess the genomic origin of epigenetic mechanisms is through detection of so-called DNA methylation quantitative trait loci (mQTL). mQTL studies aim to find associations between genetic variations and DNA methylation states using linear regression, while accounting for additive genotype effects. The extent to which genetic polymorphisms associate with variation in DNA methylation has been investigated in *Arabidopsis thaliana* (Dubin et al. 2015), maize (J. Xu et al. 2019), corals (Liew et al. 2020), red junglefowl and chicken intercrosses (Höglund et al. 2020), and humans (Gibbs et al. 2010; Heyn et al. 2013). However, we are not aware of studies that provide evidence of a genomic origin of epigenetic inheritance in wild birds (Sepers et al. 2019; Höglund et al. 2020).

Therefore, in the present study, we aim to disentangle the effects of a common origin from a common natural rearing environment on genome-wide DNA methylation in wild great tits (*Parus major*). For this, we performed a standardized partial cross-fostering experiment and assessed both CpG DNA methylation and single nucleotide polymorphism (SNP) genotypes of over 200 nestlings using a reduced representation bisulfite method (epiGBS2: Gawehns et al. 2022). Cross-fostering is a simple and highly effective technique that allowed us to separate the effects of the natural rearing environment from the brood of origin (Anholt and Mackay 2009; Winney et al. 2015). We subsequently assessed whether we could pinpoint the actual genetic polymorphism responsible for a brood of origin effect by conducting an mQTL study on the CpGs with significant brood of origin effects. Great tits offer an excellent study system for this, as previous studies on great tits have shown that DNA methylation is to some extent plastic (Viitaniemi et al. 2019; Lindner, Verhagen, et al. 2021), while on the other hand, genetic siblings show a large resemblance (Viitaniemi et al. 2019; van Oers et al. 2020). Moreover, as great tits are altricial, early development continues for a longer period of time, even after fledging, which potentially results in a higher sensitivity to naturally fluctuating conditions compared with precocial birds. These conditions vary between broods, even within the same population. Broods vary prehatching in, for example, deposited yolk hormone levels (Lessells et al. 2016; Ruuskanen et al. 2018) and posthatching in brood size (Pettifor et al. 1988), habitat quality (Naef-Daenzer and Keller 1999; Wilkin et al. 2009), parasite abundance (Allander 1998), and parental traits, such as age (Lessells et al. 2016), personality (van Oers et al. 2004; Dingemanse and Réale 2005), quality (Verhulst et al. 1995; Norte et al. 2010), and food provisioning (Wilkin et al. 2009; Pagani-Núñez and Senar 2014; van Oers et al. 2015). These developmental conditions subsequently affect nestling growth and condition (Tinbergen and Boerlijst 1990; Allander 1998; Sanz and Tinbergen 1999; Neuenschwander et al. 2003; Both et al. 2005; Pagani-Núñez and Senar 2014; Sepers 2022), various aspects of their physiology (Mertens 1969; Keller and van Noordwijk 1994; Brinkhof et al. 1999; Naef-Daenzer and Keller 1999; Naguib et al. 2011), behavior (Carere et al. 2005; Naguib et al. 2011; van Oers et al. 2015; Sepers 2022), and survival (Both et al. 2005). Using the standardized partial cross-fostering design described above, we previously reported a substantial brood of rearing effect on nestling weight, size, and behavior (Sepers 2022). Especially variance in weight was explained by the rearing environment, and this effect increased during development. These results clearly show developmental phenotypic plasticity due to across rearing-brood variation in natural environmental conditions. However, as findings on plastic responses are limited to only a handful of phenotypic changes, we expect that the majority of sites that show DNA methylation variation originate from prehatching variation, such as genetic variation as opposed to being influenced by environmental cues.

## Materials and Methods

### Subjects, Study Site, and General Procedures

We conducted the current study during the breeding season of 2018 (April to June) in the mixed wood forest of Westerheide, near Arnhem, the Netherlands (52°01′00N, 05°50′30E). The focal population of wild great tits (*P. major*) is part of a long-term nest box study, in which 228 next boxes are positioned across a 120-ha plot within the forest. To predict hatch dates, nest boxes were checked twice a week in April and May to establish initiation of nest building, first egg-laying date, clutch size, and date of start of incubation. We determined the exact hatch date, defined as the day at which the majority of eggs within a clutch hatched, by examining the broods from 2 days before the calculated hatch date until hatching.

### Cross-Fostering and Brood Size Manipulation

One to two days after hatching, clutches with the same hatch date and similar brood sizes were assigned to a cross-foster pair (hereafter CF pair). Each CF pair was randomly assigned to either the control group or the treatment group. Within each pair, a partial cross-foster design was executed as described in van Oers et al. (2015) and Sepers et al. (2021). Nestlings were weighed (digital scale, ±0.01 g) and, within their brood of origin, ranked according to their weight. Next, the nestlings were assigned to a brood by moving all the even ranked nestlings within the CF pair to one brood while moving all the odd ranked nestlings to the other brood. This approach minimized weight differences between cross-fostered (moved) nestlings and those that stayed in the brood of origin (unmoved) (van Oers et al. 2015). For control CF pairs (249 nestlings, 30 broods, and 15 CF pairs), nestlings were partially cross-fostered as described above, and the original brood size was maintained. For another study (Sepers 2022) in which we were interested in the standardized effect of brood size, brood sizes were manipulated in addition to the cross-fostering procedure for CF pairs in the treatment group. In one brood within a treatment CF pair, the original brood size was enlarged by three nestlings (159 nestlings and 14 broods), although the original brood size of the other brood was reduced by three nestlings (81 nestlings, 14 broods, and 14 CF pairs). The three nestlings were selected as random as possible, but we always aimed to keep the number of unmoved nestlings in a brood similar to the number of moved nestlings and the differences in weight between unmoved and moved nestlings minimal. Since we were interested in partitioning variation in DNA methylation into effects of the natural environmental variation and genetic origin of the nestlings, we corrected for this treatment effect (see below).

### Blood Sample Collection

To obtain methylation levels in erythrocyte DNA, blood samples were taken from the nestlings and their parents. Blood samples provide a nonlethal way of sampling DNA methylation profiles of wild individuals, and erythrocyte DNA methylation can reflect environmental effects on genes that affect the phenotype (Frésard et al. 2013). Additionally, erythrocyte DNA methylation is highly correlated with DNA methylation in brain tissue (Derks et al. 2016). DNA in avian blood consists of over 90% of erythrocyte DNA.

On days 9 to 10 after hatching, parents were caught using spring traps placed in the nest box (117 parents, 62 broods, and 31 CF pairs). The parents were sexed using breast stripe width and the presence or absence of a brood patch (Gosler 1993). Next, a blood sample of approximately 20 *µ*L was taken from the brachial vein. Half of the blood sample was stored in Queen's lysis buffer (Seutin et al. 1991), while the other half was stored in cell lysis buffer (Gentra Puregene Kit, Qiagen, USA) and kept at the NIOO-KNAW at room temperature.

On day 14 after hatching, the nestlings were taken from the nest box and ∼10 *µ*L of blood was collected from the brachial vein. We sampled 221 control nestlings from 27 broods (1 incomplete and 13 complete control CF pairs), 73 nestlings from 13 reduced broods, and 118 nestlings from 12 enlarged broods (3 incomplete and 11 complete treatment CF pairs). The samples were stored in Eppendorf tubes with 1-mL cell lysis buffer (Gentra Puregene Kit, Qiagen, USA) and kept at the NIOO-KNAW at room temperature. Nestlings were sexed using molecular markers according to the method described in Griffiths et al. (1998).

### Sample Selection and Processing

From each brood in a complete CF pair (control and treatment), we selected all four available parental samples and randomly selected the nestling samples of two or, if available, three moved and two or three unmoved nestlings. This resulted in 64 parental samples and 222 nestling samples from 20 CF pairs. Out of those 286 samples, 20 parental samples and 100 nestling samples were from 18 control broods (9 control CF pairs), 22 parental samples and 67 nestling samples were from 11 enlarged broods, and 22 parental samples and 55 nestling samples were from 11 reduced broods (11 treatment CF pairs). The nestling samples originated from 116 unmoved nestlings and 106 moved nestlings.

For sequencing, the 286 samples were pooled with 3 other samples from a different data set to generate 8 sequencing libraries, each containing 36–37 samples. One parental sample was sequenced in two libraries, as the number of reads in the initial library was low for this sample. To allow foster and origin sibling comparisons without possible effects of libraries or lanes, samples from the same CF pairs were pooled in the same library.

### epiGBS2 Library Preparation and Sequencing

We assessed genome-wide DNA methylation levels using epiGBS2 (Gawehns et al. 2022), with some improvements. Sequencing libraries were prepared at the NIOO-KNAW as described in detail in Sepers (2022). In short, DNA was isolated from 2 to 5 *µ*L blood per sample using the FavorPrep 96-Well Genomic DNA Extraction Kit (Favorgen). Subsequently, 800 ng DNA per sample was digested with *Msp*I, a restriction enzyme that recognizes and cleaves the genomic DNA at 5′-C^^^CGG sequences. We then conducted size selection by removing large fragments using beads (0.8 × AMPure XP beads). Next, the fragments were ligated to a unique barcoded adapter combination for each sample within a sequencing library, after which the fragments of all 36–37 samples were pooled into one sequencing library. The pooled fragments were exposed to sodium bisulfite, which converts unmethylated cytosines (Cs) into uracils, which will later be amplified as thymines. Bisulfite–PCR amplification was conducted with 15 PCR cycles and the KAPA HIFI Uracil + hotstart ready mix. The final amplified libraries were sequenced on an Illumina HiSeq X (150 bp, paired-end, directional) by Novogene (Novogene [HK] Company Limited, Hong Kong).

### DNA Methylation Analysis

#### Demultiplexing, Quality Control, Trimming, Alignment, and Methylation Calling

For details on demultiplexing, quality improvements, alignment, and methylation calling of the raw reads, see Sepers (2022). To assess DNA methylation levels at CpG site specifically, we used the epiGBS2 bioinformatics pipeline (Gawehns et al. 2022). This pipeline is integrated in a *Snakemake* v6.1.1 workflow (Mölder et al. 2021). Raw reads were demultiplexed, quality checked, filtered for adapter content, and merged as described for the *P. major* samples in Gawehns et al. (2022). *Cutadapt* v2.10 (Martin 2011) was used to trim the Illumina sequence and the custom part of the 3′ adapter sequence and to remove short reads (<20 bp). Raw and cleaned reads were quality checked using *FastQC* v0.11.8 (Andrews 2010), *FastQ screen* v0.11.1 in bisulfite mode (Wingett and Andrews 2018), and *MultiQC* v1.8 (Ewels et al. 2016). We aligned the cleaned reads in paired-end and nondirectional mode to the bisulfite converted and indexed *P. major* reference genome v1 (https://www.ncbi.nlm.nih.gov/assembly/GCF_001522545.3) (GCF_001522545.3) (Laine et al. 2016) using *Bismar* (https://ftp.ncbi.nlm.nih.gov/genomes/all/annotation_releases/9157/102/) v0.22.3 (Krueger and Andrews 2011) with *Bowtie* (http://bowtie-bio.sf.net/bowtie2) v2.3.5.1 (Langmead and Salzberg 2012). The mapped reads of the duplicate parental sample were merged using *SAMtools* v1.9 (Li et al. 2009). Methylation of CpGs was called in paired-end mode while removing overlap between read pairs and while ignoring the first four bp's in the R1's and R2's using *Bismark*.

#### Filtering of Methylation Calls

All subsequent steps involving R packages were performed in R v4.0.1 (R Core Team 2021). Plots were created using the R packages *BioCircos* v0.3.4 (Cui et al. 2016) and *VennDiagram* v1.60.20 (Chen and Boutros 2011).

To check if samples clustered between the eight different libraries, sexes, or groups (reduced/enlarged/control), a principal component analysis (PCA) was conducted using the raw, unfiltered data set. Additionally, a one-way analysis of variance (ANOVA) was conducted to test between any significant differences in average CpG methylation percentage between libraries. CpG site methylation was further assessed and filtered using *methylKit* v1.16.1 (Akalin et al. 2012). Reads with a coverage under 10× were excluded from the analysis, as well as those with a coverage over the 99.9 percentile to avoid PCR biases. Next, we normalized the coverage values between samples, using a scaling factor based on the median of coverage distributions. When destranding filtered data, meaning that the reads of both strands of a CpG site were merged, only CpGs that were shared between more than 80% of the chicks (*N* > 178) were included. Furthermore, we excluded CpGs which were fully methylated or fully unmethylated in all individuals.

#### Differential Methylation Analysis

To ensure that sex differences did not bias genetic or environmental effects on CpG methylation, we performed a differential methylation analysis (DMA) between the sexes to exclude CpG sites that significantly differed in DNA methylation between the sexes. However, we found no sex-specific CpG sites, and therefore, we did not perform any additional filtering steps. The *methylKit* object containing filtered CpGs was manually transformed to allow the loading in of these data in *DSS* v2.38.0 (Wu et al. 2013; Feng et al. 2014) for DMA. *DSS* assumes a beta-binomial distribution for bisulfite sequencing data and uses a dispersion shrinkage method plus a Wald statistical test to detect any differentially methylated sites (DMS) (Wu et al. 2013; Feng et al. 2014). The two-group comparison function was used in *DSS* to identify DMS between molecularly assigned females and males. A DMS was defined as having a multiple testing corrected false-discovery rate (FDR) *q* value below 0.05 and a difference in methylation percentage over 10% between the two groups.

#### Variance Partitioning

Our partial cross-fostering experimental design allowed us to compare the variability in DNA methylation profiles between genetic siblings raised in the same brood versus those raised in different broods and between foster siblings that come from the same versus a different genetic origin. To partition these variances, we constructed a linear mixed-effects model (LMM) fitted using restricted maximum likelihood estimation (REML = TRUE) with the *lme4* package v1.1 (Bates et al. 2015). The model predicted the percentage of DNA methylation observed in the nestlings at a given CpG site, including the fixed factors group (reduced/enlarged/control) and sex, and the random factors brood of origin and brood of rearing. We included group to correct for group-induced variation in brood size, leaving only natural environmental variation, including natural brood size variation. We modeled the percentage of DNA methylation instead of account for variation in coverage, as an LMM (unlike a generalized LMM [GLMM]) does not allow for modeling the dependent variable as the fraction of the number of methylated Cs over the total number of Cs. Nonetheless, an LMM can be fitted with *REML* and is therefore the preferred method. This model was run for every CpG site that passed the filtering steps (*N* = 117,521). The *P* values of the random effects were calculated using the *ranova* function of *lmerTest* v3.1.3 (Kuznetsova et al. 2017). The intraclass correlation coefficient, which measures the proportion of variation explained by the random factors, was calculated using *performance* v0.7.2. (Lüdecke et al. 2021). A significant CpG site was defined as a site with an FDR-adjusted *q* value below 0.05 and an intraclass correlation coefficient over 15%. We present the results of the model where we correct for the brood size manipulation experiment as a fixed effect. Repeating the analysis without correcting for this fixed effect minimally changed the results (see supplementary table S3, Supplementary Material online). As some nestlings were sampled on day 13 (*N* = 5) or 15 (*N* = 11) after hatching instead of on day 14 (*N* = 206), we repeated the LMM described above while including day after hatching as a fixed factor which again had minimal impacts on the results (supplementary table S3, Supplementary Material online).

### SNP Calling

We also called SNPs from epiGBS2 reads. To be able to differentiate between reads from different individuals during SNP calling, RG tags were added to the bam files that were generated during alignment. This was done using a custom Python script (v3.9.5) and the *AddOrReplaceReadGroups* function in *Picard* v2.26.5. To avoid memory issues, this was done in two batches. Next, the epiGBS2 pipeline (Gawehns et al. 2022), which has implemented *Freebayes* v1.3.2, was used for variant calling. The minimum coverage was set to three (*--min-coverage* 3). To exclude other types of polymorphisms (such as insertions or deletions), the *SplitVcfs* function and the *SNP_OUTPUT* option in *Picard* were used. Next, the two SNP files (vcf) were compressed using *bgzip* and indexed and merged using *bcftools* v1.14. If a SNP was present in both vcf files, the highest quality score was retained. The SNPs in the merged vcf file were subsequently filtered for an overall quality score of 50 or higher (*-minQ* 50), a minor allele frequency of at least 0.05 (*-maf* 0.05), and a distance of at least 30 bp apart (*-ld-window-bp-min* 30) using *vcftools* v0.1.16. To ensure that all SNPs were actual SNPs, we filtered for SNPs that were also present in a baseline list containing 8,587,616 SNPs from four whole-genome resequenced (WGS) great tits (Lindner et al. 2022). First, the baseline SNP list and the epiGBS2 SNP list were indexed using *bcftools*. Next, only SNPs for which at least 98% of the genotype was known and SNPs that were present in both lists were selected using the *bcftools* options *F_MISSING <* 0.02 and *isec*. SNPs that were in linkage disequilibrium (LD) and less than 1,000 bp apart were filtered out using the *bcftools* options *+ prune -m* 0.2 *-w* 1,000 (Smith 2020). An overview of the number of SNPs before and after filtering is given in supplementary tables S4 and S6, Supplementary Material online.

For subsequent DNA mQTL analysis, five input txt files were created using a custom Python script. The first file contained the covariates age (nestling or parent) and sex; the second file contained the genotype per SNP per individual. The genotype was either a homozygous match with the reference genome (0), a heterozygous match with the reference genome (1), or a homozygous alternative genotype (2). The third file contained the chromosome and position of each SNP, the fourth file contained the methylation percentage per CpG site per individual, and the fifth and last file contained the chromosome and start and end position of each CpG. CpGs that overlapped with a SNP were not included (see supplementary table S5, Supplementary Material online) to avoid the inclusion of false-positive methylation calls.

### mQTL Analysis

To identify SNPs which were significantly associated with the methylation percentage of CpGs for which brood of origin explained a significant part of the variance, we performed an mQTL analysis using the R package *Matrix eQTL* v2.3 (Shabalin 2012). This package tests for associations of local (*cis*) and distant (*trans*) CpG–SNP pairs, and it does so for each pair separately. We tested for associations by modeling the effect of genotype (the SNP encoded by 0, 1, and 2) on methylation percentage as additive linear and tested for its significance using *t* statistic. Included covariates were age (parent or nestling) and sex, and the covariance matrix was set to numeric() (a multiple of identity, homoskedastic, and independent errors). The maximum distance for a CpG–SNP pair to be considered *cis* was 1 million bp; otherwise, it was defined as a *trans* pair. After the analysis, *trans* associations between a SNP and CpG site that were located on the same chromosome and less than 5 million bp apart (so-called long-range *cis* mQTL) were excluded using a custom Python script. We used a Bonferroni *α*-threshold of 1.09 × 10^−07^ after correcting for 459,777 SNP–CpG tests for *cis* associations and an *α*-threshold of 4.63 × 10^−10^ correcting for 108,719,643 tests for *trans* associations.

### Gene Annotation

CpGs that had a significant variance component of brood of origin and/or brood of rearing and SNPs with a significant CpG–SNP association were annotated using the reference genome of the *P. major* v1 (https://www.ncbi.nlm.nih.gov/assembly/GCF_001522545.3). We used R packages *GenomicFeatures* v1.42.3 (Lawrence et al. 2013) and *rtracklayer* v1.50.0 (Lawrence et al. 2009) to annotate TSS regions, promoters, introns, exons, three prime untranslated region (3′UTR), and five prime untranslated region (5′UTR). We defined a TSS as being located between 300-bp upstream to 50-bp downstream of each gene's annotated starting position (Laine et al. 2016). Promoters were defined as the region 2,000-bp upstream to 200-bp downstream of the genes’ annotated starting position, which therefore overlap with the TSS (Lindner, Laine, et al. 2021). If sites were associated with both a promoter region and a TSS region, only the TSS region was reported. Upstream regions were limited to 10,000-bp upstream of the gene body, and downstream regions to 10,000-bp downstream of the gene body (Laine et al. 2016; Lindner, Verhagen, et al. 2021). When a CpG site or SNP was associated with multiple regions, we prioritized the regions in the following order: TSS, promoter, gene body (exon or intron), upstream or downstream, and intergenic (unannotated). To determine whether DNA methylation was associated with SNPs in methyltransferases, we specifically checked for the presence of significant associations involving SNPs in the methyltransferases *DNMT3A*, *DNMT3B*, and *METTLn* (*n*: 2, 4, 5, 6, 7A, 8, 9, 11B, 13, 14, 15, 16, 18, 21A, 22, 23, 24, 25, 25B, 26, and 27).

#### Gene Ontology Analysis

Enriched gene ontology (GO) terms were identified by running four GO analyses using GOrilla (Eden et al. 2009): including 1) genes with or nearby significant CpGs for brood of origin, 2) genes with or nearby significant CpGs for brood of rearing, 3) genes with or nearby SNPs involved in significant *cis* CpG–SNP associations, and 4) genes with or nearby SNPs involved in significant *trans* CpG–SNP associations. We input two lists of genes for each analysis: one of the four target lists described above, as well as a background list, consisting of all genes in which a CpG site or SNP was located (or nearby) based on our epiGBS2 data. LOC genes were excluded from GO enrichment analyses as there were too many LOC genes in the background list to check manually (1,219 and 1,473 LOC genes in the CpG and SNP background lists, respectively), and we did not want to bias the comparison between the target lists and the background list. The software was run using default parameters (species: *Homo sapiens*, single ranked list of genes, *P* < 0.001, GO database last updated on March 6, 2021). A significant GO term was defined as having an FDR-corrected *P* value below 0.05. For analyses 1 and 2, out of 9,041 genes in the background list, GOrilla recognized 3,672 and 3,609 were associated with a GO term. For analyses 3 and 4, out of all 5,255 covered genes in the background list, 4,978 were recognized by Gorilla and 4,926 were associated with a GO term.

## Results

### General Results

The number of raw and converted reads per library as well as the GC content and mapping efficiency can be found in supplementary table S1, Supplementary Material online. A total of 117,521 CpGs passed the filtering steps, representing 1.55% of all CpG dinucleotides in the great tit genome (see supplementary table S2, Supplementary Material online for remaining CpGs after each filtering step). Mean global levels of DNA methylation were 0.16% ± 6.37 × 10^−5^.

### Variance Partitioning

**FIG. 1.**
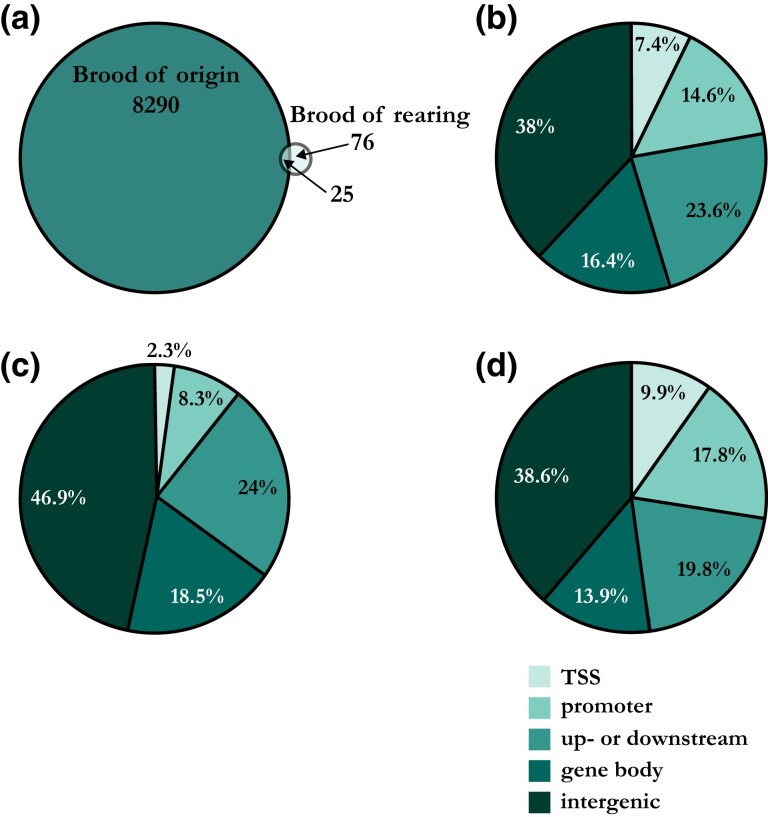
Variance in DNA methylation explained by brood of rearing and brood of origin. (*a*) Venn diagram visualizing the number of CpGs for which a significant part of variance was explained by brood of origin, rearing, or both. Pie charts visualizing (*b*) the percentages of all CpGs used in the model (*N* = 117,515) per functional region, (*c*) the percentages of significant CpGs for brood of origin per functional region, prioritized as described above, and (*d*) the percentages of significant CpGs for brood of rearing per functional region. Promoter region is excluding TSS region.

For a total of 8,315 CpGs, the variance explained by brood of origin was found to be significant and for 101 CpGs, the variance of brood of rearing. Twenty-five CpGs overlapped between these two sets (fig. 1*a*). For brood of origin, a higher percentage of sites was located in intergenic regions (46.9%) compared with the background set (38.0%); fig. 1*b* and *c*). The percentages of significant CpGs for brood of rearing generally matched the background set of sites (fig. 1*b* and *d*).

### mQTL Analysis

**FIG. 2.**
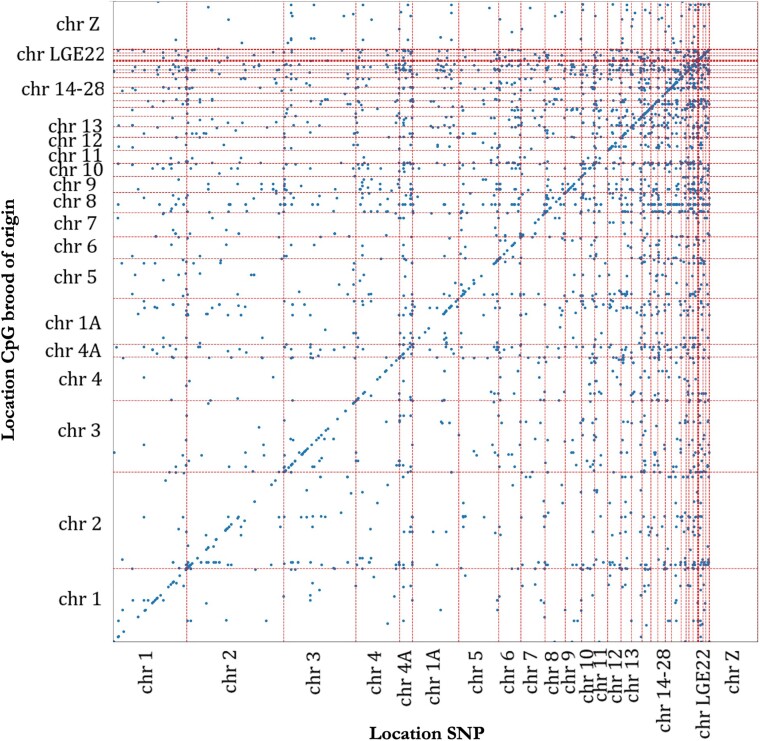
GWAS for significant origin CpGs. Each dot represents a significant SNP–CpG association at the Bonferroni-corrected 0.05 level. Each location of the SNP involved in this association is plotted against the location of the CpG within the *P. major* genome. Red dotted lines differentiate adjacently displayed chromosomes.

**FIG. 3.**
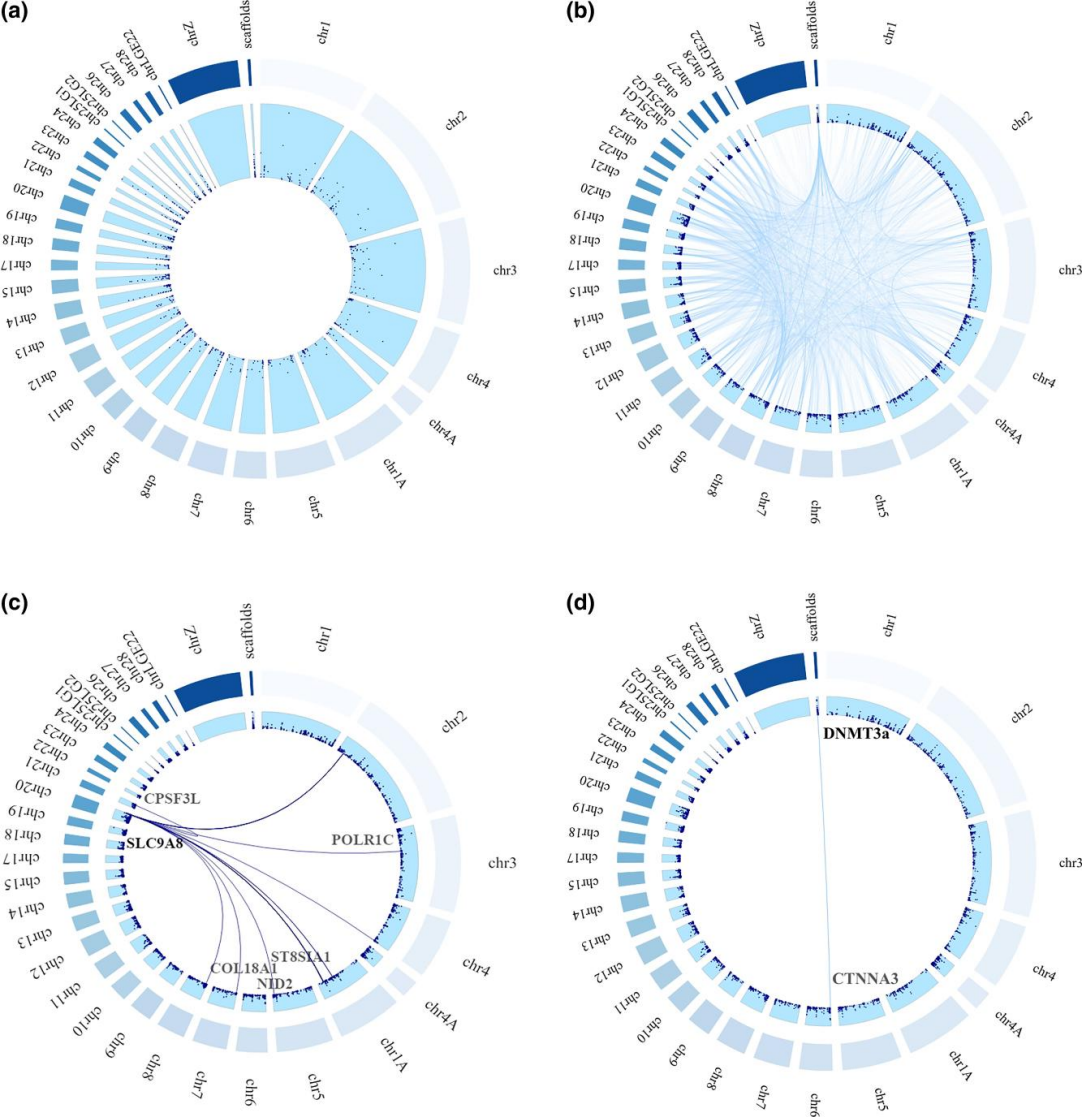
Significant *cis* and *trans* mQTLs. Circle plots showing the position of (*a*) *cis* mQTLs, (*b*) *trans* mQTLs, (*c*) *trans* mQTL hotspot in SLC9A8/OPN5-like (LOC107213364), and (*d*) *trans* mQTL in DNMT3a. Dark blue dots represent SNPs plotted against the associated location within the genome. The lines connect SNPs (gene names in black) to the different *trans* regulated CpGs (gene names in gray).

An mQTL analysis was conducted with 16,332 SNPs and 6,685 brood of origin CpGs, compromising 0.09% of CpG dinucleotides in the genome. In total, 4,956 significant associations were detected between 1,600 unique CpGs and 2,230 unique SNPs (fig. 2). Thus, from the 6,685 CpGs for which brood of origin explained a significant part of the variance in methylation, 24% of the CpGs was significantly associated with one or multiple SNPs. Out of the 4,956 associations, 754 were significant *cis* associations (all *P* < 1.09 × 10^−07^, 0.16% of all 459,777 run associations) between 680 unique CpGs and 499 unique SNPs (fig. 3*a*). We found 4,202 significant *trans* associations (all *P* < 4.63 × 10^−10^, 0.004% of all 108,719,643 run associations) between 1,145 unique CpGs and unique 1,836 SNPs (fig. 3*b*). Out of these 4,202 significant *trans* associations, 517 associations (12.30%) were with a CpG site located in a promoter region, of which 216 (5.14%) in a TSS region, and 722 (17.18%) associations were with a CpG site in a gene body. Out of the 754 significant *cis* associations, 97 associations (12.86%) were with a CpG site in a promoter region, of which 33 (4.38%) in a TSS region, and 160 (21.22%) associations included a CpG site in a gene body.

The mean distance between a *cis* mQTL and a CpG site was 150,008 bp (range: 1–990,443). Looking at different regulatory regions in more detail, the mean distance between a *cis* mQTL and a CpG site in a promoter region (including TSS), TSS, and gene body were 154,212 bp (median = 159 bp, range = 1–974,622 bp), 96,743 bp (median = 96 bp, range = 3–509,893 bp), and 155,755 (median = 1,065 bp, range = 1–990,443 bp), respectively.

One hundred and eight SNPs were significantly associated with two or more local (*cis*) CpGs and three SNPs to ten or more local CpGs. This involved a SNP in the TSS of the gene bone morphogenetic protein 3 (*BMP3*; 13 associations), a SNP in an exon of the gene mediator complex subunit 21 (*MED21*; 11 associations), and an unannotated SNP (11 associations).

Seven hundred and thirty-nine SNP sites were significantly associated with two or more distant (*trans*) CpGs and two SNPs to 20 or more distant CpGs. These so-called *trans*-hotspots involved a SNP that was situated both in an intron of the LOC gene opsin-5-like (*OPN5-like*; *LOC107213364*) and in an intron of the gene solute carrier family 9 member A8 (*SLC9A8*) (23 associations; fig. 3*c*) and a SNP upstream from the gene serine/threonine kinase 32C (*STK32C*; 20 associations).

Thirty-seven SNPs of our background list were situated in or near 10 out of 23 known methyltransferases, of which two were significantly associated with CpG methylation (table 1). Nine SNPs were located in or near *DNMT3A*, of which two (located upstream and in an intron) were significantly associated with methylation of two distant CpGs (fig. 3*d*). One of these CpGs was located in the exon of *SELPLG* and the other in an intron of *CTNNA3*.

**Table 1.** Overview and the Number of SNPs Situated in or Near Known Methyltransferases.

Methyltransferase	Number of SNPs	Significant
***DNMT3A***	**9**	**2**
***DNMT3B***	**4**	0
*METTL2*	0	0
*METTL4*	0	0
*METTL5*	0	0
*METTL6*	0	0
***METTL7A***	**2**	0
***METTL8***	**1**	0
***METTL9***	**1**	0
***METTL11B***	**2**	0
*METTL13*	0	0
*METTL14*	0	0
*METTL15*	0	0
***METTL16***	**11**	0
*METTL18*	0	0
*METTL21A*	0	0
*METTL22*	0	0
***METTL23***	**2**	0
*METTL24*	0	0
*METTL25*	0	0
*METTL25B*	0	0
***METTL26***	**2**	0
***METTL27***	**3**	0

Note.—Number of SNPs in our data set and how many of those were significantly associated with methylation of a distant CpG site. Numbers above zero are indicated in bold.

### Gene Ontology

We found five enriched GO terms for the CpGs of which a significant amount of variance was explained by brood of origin, although the terms became insignificant after FDR correction (supplementary table S7, Supplementary Material online). The terms included the biological processes negative regulation of Wnt signaling pathway (GO: 0030178, FDR = 1.00), cell–cell adhesion via plasma membrane adhesion molecules (GO: 0098742, FDR = 1.00), anatomical structure development (GO: 0048856, FDR = 1.00), and regulation of axonogenesis (GO: 0050770, FDR = 1.00) (supplementary table S7, Supplementary Material online) as well as the molecular function proximal promoter sequence-specific DNA binding (GO: 0000987, FDR = 1.00) (supplementary table S8, Supplementary Material online). Four significant GO terms were found for CpGs significant for brood of rearing. The terms included biological processes such as DNA methylation or demethylation (GO: 0044728, FDR = 6.10 × 10^−1^; GO: 0006306, FDR = 1.00), DNA modification (GO: 0006304, FDR = 8.05 × 10^−1^), and DNA alkylation (GO: 0006305, FDR = 1.00). Also, these terms did not remain significant after correcting for multiple testing (supplementary table S9, Supplementary Material online).

We detected seven enriched GO terms for the genes associated with *cis* SNPs. The GO terms were mainly related to the cell membrane and channel activity (*P* < 0.001; supplementary tables S10–S12, Supplementary Material online). For the *trans* SNPs, we detected ten enriched GO terms mainly related to cell motility, cytoskeleton, and skeletal system morphogenesis (*P* < 0.001; supplementary tables S10–S12, Supplementary Material online). However, none of the GO terms were significantly enriched after FDR correction.

## Discussion

Epigenetic modifications have been proposed to translate environmental fluctuations into heritable and adaptive phenotypic changes. However, we know relatively little about what proportion of the epigenome is regulated by genetic versus environmental factors. Using a partial cross-foster experiment, we were able to partition variance in DNA methylation into common origin and common rearing environment components in a natural population of great tits (*P. major*). Our results indicate only a small contribution of environmentally induced variation to DNA methylation early in life even though phenotypic effects, such as weight, size, and behavior, have a substantial nest of rearing effect (Sepers 2022).

### Variance Partitioning

We find that the relative contribution of erythrocyte DNA methylation that can be explained by common origin, a combination of genetic and prenatal environmental effects, by far exceeds the effect of the common rearing environment. This result is in line with studies on other vertebrates (Höglund et al. 2020), including humans (Villicaña and Bell 2021). For example, up to 35% of the variation in DNA methylation originated from additive genetic variation in three-spined stickleback (*Gasterosteus aculeatus*) crosses between individuals originating from marine and freshwater populations (Hu et al. 2021). In human twins, at least one-tenth of the CpGs had a heritability score of methylation level of over 0.5 (Grundberg et al. 2013; McRae et al. 2014; Van Dongen et al. 2016). A study on Swedish *A. thaliana* grown under two different temperature environments also showed that DNA methylation is largely genetically determined and only DNA methylation out of the CpG context showed environmental effects (Dubin et al. 2015). Our results confirm, in a natural population, that DNA methylation is a trait with limited room for plastic responses to environmental cues during early developmental stages, at least in this vertebrate study system.

A pronounced genetic effect on variation DNA methylation implies that the heritability of epigenetic marks is favored over epigenetic plasticity. Plasticity might be favored in a fluctuating environment, where levels of predictability (variability between generations) are low, but genetic assimilation (the process where an environmentally induced phenotype becomes genetically encoded) should be favored when environmental conditions are predictable and reliable (Angers et al. 2010). Alternatively, if a particular SNP or a combination of SNPs controls the sensitivity to gain alternative methylation states of a given CpG site depending on the environment (either in *cis* or in *trans*), this allele might increase in frequency over generations, which will lead to an increase in sensitivity to environmental fluctuations. The methylation marks are not inherited themselves, but the genes important for plasticity in response to environmental cues are. This plasticity can be regarded as an advantage itself, as opposed to the result of the plasticity (Angers et al. 2010). This so-called Baldwin effect (Simpson 1953; Crispo 2007), as described in Angers et al. (2010), might be more beneficial as opposed to a strict genetic control of DNA methylation only, given that the environments between generations are heterogeneous or unpredictable (West-Eberhard 2003; Jensen 2013). Our results suggest that, at least during early development, a genetic rather than environmental source of epigenetic variation is favored.

### Methylation Quantitative Trait Loci

Using the subset of CpGs that were significantly affected by brood of origin in a subsequent mQTL analysis, we revealed candidate SNPs that underlie variation in DNA methylation. In addition, we identified SNPs that were both associated in *cis* and *trans* with the CpGs. From the 6,685 CpGs for which brood of origin explained a significant part of the variance in methylation and that did not overlap with a SNP, 24% of the CpGs (1,600 unique CpGs) were significantly associated with one or multiple SNPs. Comparing our results to findings on other study systems (Gibbs et al. 2010; Heyn et al. 2013; Dubin et al. 2015; X. Xu et al. 2019; Höglund et al. 2020), we find a higher percentage of CpGs that are significantly associated with a SNP. This difference is likely to be caused by the fact that we used a preselected set of CpGs when conducting our mQTL analysis. Overall, we find that genetic variation does indeed explain variation in DNA methylation.

We identified 754 *cis* associations, which suggest that local genetic variation directly affects CpG methylation and possibly indirectly affects gene expression (Gutierrez-Arcelus et al. 2013). The genes associated with *cis* SNPs were mainly related to the cell membrane and channel activity. *Cis* relationships therefore seemingly play a general role in cell–cell communication during development. Whether these are specific for early development is not clear and could be investigated by conducting similar studies at different ages.

Several SNPs appeared to influence DNA methylation state at not just one but multiple different CpGs. Specifically, we found two small *trans*-acting hotspots, involving SNPs that were significantly associated with methylation of 20 or more distant CpGs. One of these SNPs was situated in an intron of the gene *SLC9A8* (also known as *NHE8*) and in an intron of the gene *OPN5-like* (*LOC107213364*). *SLC9A8* is involved in pH regulation (Nakamura et al. 2005), which is important for, for example, retinal function (Jadeja et al. 2015). *OPN5* is a photoreceptor important for entrainment of the circadian clock and has been linked to sexual maturation in several avian species (Stevenson and Ball 2012; Banerjee et al. 2018; Hanlon et al. 2021; Van Wyk and Fraley 2021). More SNPs that were significantly associated with CpG methylation appeared to be in or near genes associated with reproduction, maturation, and growth. Several of these SNPs were in or near myosin light-chain genes (*MYLK2*, *MYL2*, and *MYL3*). Genes related to the biosynthesis of myosin are involved in egg laying (Kupittayanant et al. 2009; Liu et al. 2018), and differential methylation of these genes has been related to growth in broiler chickens (Hu et al. 2013) and the onset of reproduction in the great tit (Lindner, Laine, et al. 2021). The above-described genes and part of their functions are supported by the (although not significantly) enriched GO term skeletal system morphogenesis. Therefore, these results indicate that the identified SNPs genetically control (sexual) maturation, development, and growth via DNA methylation. However, causal relationships between SNPs, DNA methylation, gene expression, and phenotypic traits remain to be established. We did not find large *trans*-acting methylation hotspots as in the study of Höglund et al. (2020), where in red junglefowl and chicken intercrosses, almost half of the *trans* mQTL were associated with only five loci (Höglund et al. 2020). This is also likely caused by the limited number of SNPs we used on our data set.

The second small *trans*-acting hotspots involved a SNP upstream from the gene *STK32C* which is highly expressed in the brain and has been linked to, for example, Alzheimer's disease (Gasparoni et al. 2018; Lin et al. 2020) and depression (Dempster et al. 2014). More SNPs that were significantly associated with CpG methylation appeared in or near genes associated with neurological diseases, cognition, or behavior. Three SNPs in an exon(s) or downstream of the dopamine receptor D2 (*DRD2*) are associated with methylation in three *trans* cases and one *cis* case. *DRD2* methylation has been linked to personality in chimpanzees (Staes et al. 2022), and *DRD2* polymorphisms have been linked to (putative) personality traits in humans (Smillie et al. 2010). Furthermore, associations between exploratory behavior and a polymorphism in *DRD4*, a gene in the same family as *DRD2*, have been detected in the great tit (Fidler et al. 2007; Timm et al. 2019), and variation in great tit personality might also be mediated by *DRD4* methylation (Riyahi et al. 2015; Verhulst et al. 2016). These links with neurological diseases and personality indicate a role for (at least partially) genetically controlled DNA methylation patterns in the regulation of behavior. Although we previously did not find evidence that DNA methylation explained heritable variation in personality in great tits (van Oers et al. 2020), it is important to note that personality is polygenic (Santure et al. 2015) and likely affected by genotype by environment interactions (van Oers et al. 2005). Thus, the *DRD2* polymorphisms might not have been present in the individuals in our previous study, whereas other polymorphisms that explain variation in personality (but did not affect DNA methylation) were. However, this remains speculative.

In addition to these hotspots, we found SNPs in or near *KLF8* and several DexD-box helicases (*DDX23* and *DDX54*) that were associated with methylation of one or more local or distant CpGs. *DDXn* genes and *KRF8* are important for transcription regulation and RNA metabolism (van Vliet et al. 2000; Fuller-Pace 2006). Interestingly, these genes and the myosin light-chain genes described above overlap in terms of gene family or function with the genes described in an mQTL study conducted on red junglefowl and chicken intercrosses (Höglund et al. 2020). In that study, *MYLK*, *KLF12*, and *DDX18* were putatively casually linked to methylation and gene expression in red junglefowl and chicken intercrosses, indicating that these SNPs underlie a domesticated phenotype (Höglund et al. 2020). We here show that these genes are also important for explaining natural variation in DNA methylation.

Although we did not find major *trans* associations in or near the majority of methyltransferases that were covered by our SNP list, we did find significant SNP–CpG associations involving *DNMT3A*. *DNMT3A* is encoded by the gene *DNMT3A* and, at least in mammals, carries out de novo DNA methylation. Furthermore, it has nonselective and ubiquitous activity and is essential for early development (Okano et al. 1998, 1999) and cell differentiation (Challen et al. 2012; Wu et al. 2012). Nine SNPs were in or near *DNMT3A*, of which two (located upstream and in an intron) were significantly associated with methylation of two distant CpGs. One of these CpGs was located in an exon of the gene *SELPLG*, and the other was located in an intron of the gene *CTNNA3*. Both genes are involved in cell–cell adhesion. Previous studies have highlighted the role for *SELPG* and cadherin-associated genes for cell differentiation and fibrosis. In *DNMT3A* and *DNMT3B* knockout experiments, another cadherin-associated gene (*CTNNB1*) was hypomethylated and overexpressed (Challen et al. 2014). *DNMT3A* was found to be hypermethylated and underexpressed in systemic sclerosis microvascular endothelial cells, whereas *CTNNA1* was found to be hypomethylated (Nada et al. 2022). Furthermore, epigenetic dysregulation of both *DNMT3A* and *SELPG* is associated with systemic sclerosis (Tsou et al. 2019). We therefore conclude that CpG variation caused by genomic variation in methyltransferases is pointing toward variation in general preprogrammed genetic differences in early developmental processes.

We did not find significant associations involving SNPs in *METTLs*, which suggest that *METTLs* are more important for RNA and protein modifications than for DNA modifications, at least during early development (for an overview, see Wong and Eirin-Lopez 2021). As we did not find major *trans* associations in or near the majority of methyltransferases, our results might also imply that *DNMTs* and *METLLs* are essential for normal development and functioning via regulation of DNA methylation to such a degree that their sequence and function have been highly conserved (Wong and Eirin-Lopez 2021). Any genetic variation could be lethal (see Lyko 2018), and as a result, no quantitative variation between individuals might be expected. Although catalyzation of DNA methylation seems to be a very conserved and stable process, demethylation might be more flexible. Demethylation is catalyzed by enzymes of the 10–11 translocation (*TET*) family (Tahiliani et al. 2009; Ito et al. 2011; Okuzaki et al. 2017). As studies on active demethylation have mainly focused on zebrafish and mammals (for an overview, see Law and Jacobsen 2010) and hardly on birds (Okuzaki et al. 2017), the degree of sequence and function variation in member of the *TET* family during nestling development remains largely elusive.

When observing the results from the mQTL analysis, many significant associations involved SNPs near the ends of chromosomes. This can be explained by the fact that we excluded SNPs that were in LD, that is, nonrandomly associated. LD is not evenly distributed across the genome as LD is lower near the ends of chromosomes (Stapley et al. 2010), and as a result, we excluded more SNPs at the center than those at the ends of chromosomes. The uneven distribution of LD is probably due to the uneven distribution of recombination within bird chromosomes. Recombination rates are often higher at the telomeric regions than those at the center of chromosomes (Stapley et al. 2008; Backström et al. 2010; Stapley et al. 2010; van Oers et al. 2014). As recombination is expected to break down LD, this probably explains lower LD near the ends of chromosomes.

In our study, we did not find associations between the remaining 69% of analyzed CpGs and a SNP. There are several possible explanations for that. First, it is possible that DNA methylation on the remaining CpGs is explained by a common environmental effect occurring before cross-fostering rather than by a genetic polymorphism. Alternatively, since epiGBS2 only targets a fraction of the genome and we therefore only included 1.97% of all known true SNPs, it is likely that variation in many other CpGs is indeed associated with a SNP that was not included in the analyses. Given the strict filtering process, the number of significant associations and the number of sites for which methylation is explained by genetic variation are most likely underestimated. However, since we were interested in identifying high-quality candidates, we used these strict selection criteria for including SNPs in our analyses. Another plausible explanation might be that variation in these sites is explained by genetic polymorphisms other than SNPs, such as copy number variations (da Silva et al. 2018) or tandem repeats (Gemayel et al. 2010). Finally, a logical explanation is that some nonsignificant CpG–SNP associations were the result of type II errors. These true associations might not have been picked up because of limited statistical power. Regardless, the fact that 24% of the CpGs are associated with a SNP in our heavily filtered list and after strict Bonferroni correction confirms our expectation that DNA methylation is highly genotypically controlled.

### SNP Calling with epiGBS2

To our knowledge, this is the first study that uses the updated epiGBS2 pipeline to obtain both DNA methylation and variant information from epiGBS2 data in a vertebrate species (Gawehns et al. 2022), where only one study used the former epiGBS version (Meröndun et al. 2019). The number of SNPs that can be called in the available pipeline highly depends on the downstream analysis. A study that validated SNP calling from WGBS data found that 12% to 31% of all known SNPs were also identifiable in WGBS data when using EpiFreebayes (Lindner et al. 2022). As epiGBS2 is a reduced representation technique, it covers roughly 3% of the genome when the restriction enzyme *Msp*I is used. Therefore, we would expect to identify at best 0.93% (31% * 0.03) of all true SNPs. However, 1.97% (169,032 SNPs) of all known SNPs were also identifiable in our epiGBS2 data set, which is higher than expected.

By only including quality filtered epiGBS2 SNPs that are also included in the baseline SNP list, we omitted half of the epiGBS2 SNPs (164,806 SNPs). The loss of SNPs is at least partly explained by incompleteness of the baseline SNP list derived from only four female great tits. Since the epiGBS2 data set was based on 286 individuals and the chance of picking up genetic variation increases with sample size, we likely picked up new genuine SNPs. A reason for being very strict in selecting SNPs for our mQTL analysis is that part of the epiGBS2 pipeline is likely to consist of false positives. SNP calling with bisulfite-treated data is challenging as true cytosine to thymine SNPs have to be differentiated from unmethylated Cs that appear as thymines due to the chemical treatment (Lindner et al. 2022). Methylated Cs are known to be prone to mutation to a thymine (Coulondre et al. 1978; Pértille et al. 2019), and in humans, 63% of SNPs in CpGs in CpG islands are C to T transitions (Tomso and Bell 2003). Therefore, this is an important source of error. As part of the epiGBS2 pipeline, this should be dealt with by the double-masking method (Nunn et al. 2022). This preprocessing step converts nucleotides in bisulfite context to the corresponding nucleotide in the reference genome, and nucleotides which may have arisen as a result of the bisulfite treatment are given a base quality score of 0. Despite the double-masking method, EpiFreebayes and other SNP calling tools are known to be most sensitive to thymine to cytosine and adenine to guanine false-positive SNPs (Lindner et al. 2022).

We did encounter not only false-positive SNPs but also false-positive methylation calls. A total of 1,630 out of 8,315 origin CpGs (17%) overlapped with a SNP. As we used SNPs called from whole-genome resequencing data to verify the SNPs called from the epiGBS2 data, we are confident that the SNPs are true SNPs and that some true cytosine to thymine and guanine to adenine SNPs were most likely misidentified as unmethylated Cs during methylation calling (Heckwolf et al. 2020). At least part of the false-positive SNP and methylation calls is due to nondirectional alignment of the reads in this study. Therefore, the data were treated as if it was unknown where a read originated from, and all four strands (original top, original bottom, complementary to original top, and complementary to original bottom) could produce valid alignments. Nondirectional mapping will increase the mapping efficiency as compared with directional mapping, but it will also introduce more alignment errors and more incorrect methylation and SNP calls (Laine et al. 2022). As epiGBS2 is a (although not conventual) directional protocol, we recommend to align future epiGBS2 data in directional mode as is done in the epiGBS2 pipeline (Gawehns et al. 2022). This was not feasible with the current data set. Furthermore, we recommend to check bisulfite-treated data for false-positive methylation calls if a SNP list is available.

### Limitations and Future Studies

Despite a carefully designed and executed experimental setup, we want to point out potential improvements for future studies. In the current study, a common origin determined methylation variation at more CpGs compared with a common environment. Still, a large proportion of the variation remains unexplained, and for a large proportion of the CpGs, we did not find a common origin or a common rearing environment effect. This is likely due to the limited power of current bisulfite methods (Laine et al. 2022). Nevertheless, this does not affect the relative chance of finding genetic or environmental effects on variation in DNA methylation.

Although our experimental design allowed us to separate genetic versus environmental effects to a large extent, the fact that cross-fostering happened on day 1 or 2 means that variation of common origin can consist of environmental effects that the nestlings experienced up until cross-fostering and true genetic effects. This includes the environmental effects that the gametes of the parents experienced, effects within the egg, and influences experienced on day 1 or 2 posthatching. In addition, our study design focused on methylation during early life only (until day 14 posthatching). Future studies should experimentally change early environment at an earlier stage, for example, by manipulating the egg environment with injecting hormones and also investigate whether the relative influence of genes and environment changes over a lifetime.

Due to the restriction enzyme used in epiGBS2 (*Msp*I), we enrich our sequences heavily on CG-rich areas and therefore focus mainly on CpG islands. In a study on genome-wide human brain CpG methylation, it was concluded that quantitative trait loci occurred with a higher likelihood for CpGs outside of CpG islands (Gibbs et al. 2010). This indicates that we are likely very conservative in the number of genetic associations we present here.

Furthermore, we focused in our study on the link between genomic variation in genes and CpG methylation. However, the association between genetic and epigenetic variation can strongly depend on the genomic region. For example, in *A. thaliana*, CHH methylation on transposons was highly associated with genetic variation (Dubin et al. 2015). A likely role for DNA methylation on transposable element activity was already identified in another study on great tits (Derks et al. 2016). Expanding our analysis to include transposable elements is therefore a promising avenue.

Lastly, our study focused on CpG methylation in DNA originating from red blood cells, as this allowed us to obtain information on DNA methylation at different time points. Furthermore, studies on great tits have previously found a significant positive correlation between DNA methylation as measured in blood compared with DNA methylation in brain (Derks et al. 2016) and liver tissue (Lindner, Verhagen, et al. 2021). These correlations are present at the among-gene level, but whether this also holds for associations between variation in gene expression and DNA methylation needs to be verified. This indicates the need for more validation steps to examine how environmentally induced changes in methylation (nest of rearing sites) relate to changes in gene expression among various tissues and, consequently, the phenotype. Targeted studies are needed to determine if epigenetic mechanisms causally affect gene expression tissue specifically and how these changes in gene expression also affect the phenotype.

### Conclusion

Epigenetic variation that is induced during early development has been suggested to affect the life-long expression of phenotypic traits and if inherited could potentially contribute to soft selection (Richards 2006). We here show that the relative contribution of the early rearing environment to variation in DNA methylation in 14-day-old nestling songbirds is extremely small when compared with the genetic contribution. To our knowledge, this is the first study that has shown evidence of genomic variation underlying epigenetic variation in a wild bird species. This implies that DNA methylation is a mechanism that mostly acts in dependence of genetic variation, and therefore, we conclude that it is unlikely that DNA methylation acts as a mechanism underlying soft selection. Although our study did not assess functional causality between the SNPs and CpG methylation, the putative functions of the genes that we here identified to associate with epigenetic variation point to a genetic basis of variation in growth, (sexual) maturation, and early development. This indicates that DNA methylation variation of developing vertebrates is likely an obligatory mark (Richards 2006) translating genetic variation into gene expression, rather than being a plastic mechanism to adapt quickly to environmental changes (Kilvitis et al. 2014). Intergenerational patterns of CpG methylation are therefore likely caused by common genetic factors rather than resembling an acquired trait (Heard and Martienssen 2014). To better understand the potential evolutionary role of environmentally induced and genetically inherited methylation marks, a better understanding of the relative proportion of the respective epialleles (Feil and Fraga 2012), as well the functionality of the genes affected, is required.

## Supplementary Material

msad086_Supplementary_Data

## Data Availability

The raw genomic data sets are deposited on NCBI under BioProject ID PRJNA208335. The multiplexed reads are available under the SRA accessions SRX18523280, SRX18523281, SRX18523282, SRX18523283, SRX18523284, SRX18523285, SRX18523286, and SRX18523287 before publication. The epiGBS2 bioinformatics pipeline can be accessed on Github (https://github.com/nioo-knaw/epiGBS2). All scripts and codes used for further analysis are available at https://github.com/rshuhuachen/great-tit-methylation-genes-environment.
