# Supplementary material for: Variation in DNA Methylation in Avian Nestlings Is Largely Determined by Genetic Effects
Source: Mol Biol Evol. 2023 Apr 11;40(4):msad086. doi: 10.1093/molbev/msad086 (PMC10139706; doi:10.1093/molbev/msad086)
Supplement: msad086_Supplementary_Data [file msad086_supplementary_data.docx]

Supplementary information to

Variation in DNA methylation in avian nestlings is largely determined by genetic effects

Bernice Sepers^*^, Rebecca Shuhua Chen^,*^, Michelle Memelink, Koen J.F. Verhoeven and Kees van Oers

**These authors contributed equally*

| ***Supplementary Table 1****. Summary statistics per epiGBS2 library.* | | | | | | | |
| --- | --- | --- | --- | --- | --- | --- | --- |
| **Library** | **# raw reads R1** | **# converted reads** | **% converted reads** | **# non-fully converted reads** | **% non-fully converted reads** | **% GC content (mean R1, R2)** | **Mapping efficiency** |
| **1** | 8,132,822 | 7,778,542 | 95.64 | 354,279 | 4.36 | 35 | 48.48 |
| **2** | 7,582,585 | 7,315,951 | 96.48 | 266,634 | 3.52 | 34 | 43.64 |
| **3** | 6,871,417 | 6,640,273 | 96.64 | 231,144 | 3.36 | 34 | 46.57 |
| **4** | 8,327,338 | 8,064,191 | 96.84 | 263,146 | 3.16 | 34 | 45.68 |
| **5** | 7,638,997 | 7,418,748 | 97.12 | 220,248 | 2.88 | 34 | 47.31 |
| **6** | 7,841,377 | 7,455,892 | 95.08 | 385,486 | 4.92 | 34 | 45.14 |
| **7** | 5,885,571 | 5,654,890 | 96.08 | 230,681 | 3.92 | 33 | 47.49 |
| **8** | 6,536,573 | 6,316,517 | 96.63 | 220,056 | 3.37 | 34 | 47.22 |

| ***Supplementary Table 2****. Number of CpGs after each filtering step.* | | | |
| --- | --- | --- | --- |
| **CpGs before filtering** | **CpGs after 10X and 99.9 percentile filtering** | **CpGs after 0% or 100% methylation filtering and uniting** | **Shared CpGs after uniting** |
| 2,768,598 | 2,070,830 | 117,525 | 117,521 |

| ***Supplementary Table 3****. Number of CpGs for which the brood of origin and brood of rearing explained a significant part of the variance. To assess an effect of day after hatching on which the nestlings were sampled, a linear mixed model with day (after hatching) (model 2) was compared to the model we chose in our analyses (model 1). To assess the effect of brood size manipulation treatment, a linear mixed model without treatment was used (model 3). The two models were run for each of the 117,521 CpGs.* | | |
| --- | --- | --- |
| **Model** | **Brood of origin** | **Brood of rearing** |
| Model 1: methylation level ~ treatment + sex + (1\|origin) + (1\|rearing) | 8,315 | 101 |
| Model 2: methylation level ~ treatment + sex + day + (1\|origin) + (1\|rearing) | 8,312 | 102 |
| Model 3: methylation level ~ sex + (1\|origin) + (1\|rearing) | 8,527 | 106 |
| Significant in model 1 but not in model 2 | 290 | 2 |
| Significant in model 2 but not in model 1 | 287 | 3 |
| Significant in model 1 but not in model 3 | 165 | 6 |
| Significant in model 3 but not in model 1 | 371 | 11 |

| ***Supplementary Table 4****. Number of SNPs called with epiGBS2 including subsequent filtering steps.* | |
| --- | --- |
| **Filter step** | **# SNPs** |
| Called with epiGBS2 (coverage ≥ 3) | 7,152,397 |
| QC ≥ 50, MAF ≥ 0.05, distance ≥ 30 bp | 333,838 |
| Baseline SNP list (WGS) | 169,032 |
| known genotype ≥ 98%, LD 0.2, distance ≥ 1000 bp | 16,332 |

| ***Supplementary Table 5****. Number of origin CpGs that are left after checking for overlap with one of the 16,332 true SNPs.* | |
| --- | --- |
| **Filter step** | **# origin CpGs** |
| Significant | 8,315 |
| Cytosine 0 bp distance from SNP | *7,447* |
| Cytosine -1 bp distance from SNP | 6,685 |

| ***Supplementary Table 6****. Number of cis and trans CpG-SNPs associations, including subsequent filtering steps. The mQTL analysis was done with 6,685 origin CpGs and 16,332 SNPs.* | | | |
| --- | --- | --- | --- |
| **Filter step** | | **# cis associations** | **# trans associations** |
| Distance  CpG-SNP pair: | ≤ 1,000,000 bp cis  > 1,000,000 bp trans | 459,777 | 108,719,643 |
| Different chromosome or distance ≥ 5 million bp | | - | 107,955,112 |
| P-value: | < 1.09 x 10^-07^ cis  < 4.63 x 10^-10^ trans | 754 (680 unique CpGs and 499 unique SNPs) | 4,202 (1,145 unique CpGs and unique 1,836 SNPs) |
| Cis and trans combined | | 4,956 significant cis and trans associations  (1,600 unique CpGs and 2,230 unique SNPs) | |

| ***Supplementary Table 7****. Enriched GO terms for the ontology biological process with significant FDR q-values for all the genes associated with CpG significant for brood of origin. The number of genes in the target list annotated to the particular GO category and the number of genes in the background list annotated to a certain GO category are shown in the B and b columns, respectively. The total number of (recognized) genes was 5,445 in the brood of origin target list and the total number of genes in the background list was 9,041.* | | | | | | | |
| --- | --- | --- | --- | --- | --- | --- | --- |
| **GO Term** | **Description** | **P-value** | **FDR q-value** | **Enrichment** | **B** | **b** |  |
| GO:0030178 | negative regulation of Wnt signaling pathway | 1.62 x 10^-4^ | 1.00 | 1.58 | 57 | 38 |  |
| GO:0098742 | cell-cell adhesion via plasma-membrane adhesion molecules | 7.96 x 10^-4^ | 1.00 | 1.58 | 45 | 30 |  |
| GO:0048856 | anatomical structure development | 8.48 x 10^-4^ | 1.00 | 1.12 | 807 | 381 |  |
| GO:0050770 | regulation of axonogenesis | 8.60 x 10^-4^ | 1.00 | 1.56 | 47 | 31 |  |

| ***Supplementary Table 8****. Enriched GO terms for the ontology molecular function with significant FDR q-values for all the genes associated with CpG significant for brood of origin. The number of genes in the target list annotated to the particular GO category and the number of genes in the background list annotated to a certain GO category are shown in the B and b columns, respectively. The total number of (recognized) genes was 5,445 in the brood of origin target list and the total number of genes in the background list was 9,041.* | | | | | | |
| --- | --- | --- | --- | --- | --- | --- |
| **GO Term** | **Description** | **P-value** | **FDR q-value** | **Enrichment** | **B** | **b** |
| GO:0000987 | proximal promoter sequence-specific DNA binding | 9.33 x 10^-4^ | 1.00 | 1.22 | 256 | 133 |

| ***Supplementary Table 9****. Enriched GO terms for the ontology biological process with significant FDR q-values for all the genes associated with CpG significant for brood of rearing. The number of genes in the target list annotated to the particular GO category and the number of genes in the background list annotated to a certain GO category are shown in the B and b columns, respectively. The total number of (recognized) genes was 133 in the brood of rearing target list and the total number of genes in the background list was 9,041.* | | | | | | |
| --- | --- | --- | --- | --- | --- | --- |
| **GO Term** | **Description** | **P-value** | **FDR q-value** | **Enrichment** | **B** | **b** |
| GO:0044728 | DNA methylation or demethylation | 5.72 x 10^-5^ | 6.10 x 10^-1^ | 17.19 | 12 | 4 |
| GO:0006304 | DNA modification | 1.51 x 10^-4^ | 8.05 x 10^-1^ | 13.75 | 15 | 4 |
| GO:0006306 | DNA methylation | 5.40 x 10^-4^ | 1.00 | 17.19 | 9 | 3 |
| GO:0006305 | DNA alkylation | 5.40 x 10^-4^ | 1.00 | 17.19 | 9 | 3 |

| ***Supplementary Table 10****. Enriched GO terms for the ontology cellular component with significant FDR q-values for all the genes associated with a cis or trans SNP. The number of genes in the target list annotated to the particular GO category and the number of genes in the background list annotated to a certain GO category are shown in the B and b columns, respectively. The total number of (recognized) genes was 308 in the cis target list, 1067 in the trans target list and the total number of genes in the background list was 4926.* | | | | | | |
| --- | --- | --- | --- | --- | --- | --- |
| **GO Term** | **Description** | **P-value** | **FDR q-value** | **Enrichment** | **B** | **b** |
| ***Cis*** | | | | | | |
| - | - | - | - | - | - | - |
| ***Trans*** | | | | | | |
| GO:0030864 | cortical actin cytoskeleton | 3.16 x 10^-4^ | 4.85 x 10^-1^ | 2.49 | 26 | 14 |
| GO:0030863 | cortical cytoskeleton | 3.62 x 10^-4^ | 2.78 x 10^-1^ | 2.31 | 32 | 16 |
| GO:0005856 | cytoskeleton | 8.69 x 10^-4^ | 4.45 x 10^-1^ | 1.33 | 341 | 98 |

| ***Supplementary Table 11****. Enriched GO terms for the ontology molecular function with significant FDR q-values for all the genes associated with a cis or trans SNP. The number of genes in the target list annotated to the particular GO category and the number of genes in the background list annotated to a certain GO category are shown in the B and b columns, respectively. The total number of (recognized) genes was 308 in the cis target list, 1067 in the trans target list and the total number of genes in the background list was 4926.* | | | | | | |  |
| --- | --- | --- | --- | --- | --- | --- | --- |
| **GO Term** | **Description** | **P-value** | **FDR q-value** | **Enrichment** | **B** | **b** | |
| ***Cis*** | | | | | | |  |
| GO:0005509 | calcium ion binding | 1.64 x 10^-4^ | 4.82 x 10^-1^ | 2.10 | 206 | 27 | |
| ***Trans*** | | | | | | |  |
| GO:0005044 | scavenger receptor activity | 4.81 x 10^-4^ | 1.00 | 2.89 | 16 | 10 | |

| ***Supplementary Table 12****. Enriched GO terms for the ontology biological process with significant FDR q-values for all the genes associated with a cis or trans SNP. The number of genes in the target list annotated to the particular GO category and the number of genes in the background list annotated to a certain GO category are shown in the B and b columns, respectively. The total number of (recognized) genes was 308 in the cis target list, 1067 in the trans target list and the total number of genes in the background list was 4926.* | | | | | | |
| --- | --- | --- | --- | --- | --- | --- |
| **GO Term** | **Description** | **P-value** | **FDR q-value** | **Enrichment** | **B** | **b** |
| ***Cis*** |  |  |  |  |  |  |
| GO:0051051 | negative regulation of transport | 2.14 x 10^-5^ | 2.54 x 10^-1^ | 2.71 | 124 | 21 |
| GO:0033194 | response to hydroperoxide | 2.03 x 10^-4^ | 1.00 | 10.66 | 6 | 4 |
| GO:0050915 | sensory perception of sour taste | 2.42 x 10^-4^ | 9.59 x 10^-1^ | 15.99 | 3 | 3 |
| GO:0032501 | multicellular organismal process | 2.57 x 10^-4^ | 7.63 x 10^-1^ | 1.39 | 1062 | 92 |
| GO:1901380 | negative regulation of potassium ion transmembrane transport | 4.51 x 10^-4^ | 1.00 | 9.14 | 7 | 4 |
| GO:0071709 | membrane assembly | 5.08 x 10^-4^ | 1.00 | 6.66 | 12 | 5 |
| ***Trans*** | | | | | | |
| GO:0051270 | regulation of cellular component movement | 5.15 x 10^-5^ | 6.12 x 10^-1^ | 1.39 | 369 | 111 |
| GO:0040012 | regulation of locomotion | 1.96 x 10^-4^ | 1.00 | 1.35 | 368 | 108 |
| GO:2000145 | regulation of cell motility | 2.19 x 10^-4^ | 8.68 x 10^-1^ | 1.37 | 341 | 101 |
| GO:0030334 | regulation of cell migration | 3.51 x 10^-4^ | 1.00 | 1.36 | 325 | 96 |
| GO:0048705 | skeletal system morphogenesis | 4.48 x 10^-4^ | 1.00 | 1.99 | 51 | 22 |
| GO:0032879 | regulation of localization | 8.93 x 10^-4^ | 1.00 | 1.18 | 931 | 238 |
